# Current Therapeutic Strategies, Recent Advances and the Emerging Potential of Natural Products in Hepatocellular Carcinoma

**DOI:** 10.1155/sci5/3431270

**Published:** 2026-06-09

**Authors:** Felix Wambua Muema, Catherine M. Miller, Lionel Hebbard, Phurpa Wangchuk

**Affiliations:** ^1^ College of Science and Engineering, James Cook University, Cairns Campus, Cairns, 4878, Queensland, Australia, health.qld.gov.au; ^2^ Australian Institute of Tropical Health, and Medicine, James Cook University, Cairns Campus, Cairns, 4878, Queensland, Australia, health.qld.gov.au; ^3^ College of Medicine and Dentistry, James Cook University, Smithfield, 4878, Queensland, Australia, health.qld.gov.au; ^4^ Department of Molecular Genetics, Centre for Molecular Therapeutics, James Cook University, Townsville, 4811, Queensland, Australia, health.qld.gov.au; ^5^ Storr Liver Centre, Westmead Institute for Medical Research, Westmead Hospital and University of Sydney, Westmead, 2145, New South Wales, Australia, health.qld.gov.au

**Keywords:** cancer, drug discovery, hepatocellular carcinoma, natural products, phytochemicals

## Abstract

Hepatocellular carcinoma (HCC) accounts for 75%–85% of all primary liver cancers and remains one of the leading causes of cancer‐related mortality worldwide. Despite advances in diagnosis and systemic therapies, the clinical management of HCC is hindered by limited therapeutic efficacy, drug resistance, systemic toxicity and high recurrence rates. Consequently, there is a pressing need for alternative and complementary treatment strategies. Natural products, particularly plant‐derived phytochemicals, have long been valued for their diverse pharmacological activities and represent a promising source of novel anticancer agents. This review critically examines current HCC treatment modalities and their limitations, while highlighting the therapeutic potential of phytochemicals based on preclinical and clinical evidence, with emphasis on their molecular mechanisms of action. Furthermore, recent advances in nanotechnology‐based drug delivery systems are discussed as a potential way to overcome key translational barriers associated with phytochemicals, including poor bioavailability, limited tumour selectivity and suboptimal pharmacokinetics. The integration of phytochemistry with nanomedicine offers a powerful strategy to enhance therapeutic efficacy, reduce systemic toxicity and improve clinical translation. We identify apoptosis induction, suppression of PI3K/Akt, MAPK, STAT3, NF‐κB and HIF‐1 signalling, and modulation of oxidative stress and metastasis as the dominant anti‐HCC mechanisms of major phytochemicals. In addition, we highlight nanotechnology‐based delivery systems as a key translational strategy to improve solubility, tumour selectivity and therapeutic performance of these compounds. Collectively, this review underscores the promise of plant‐derived compounds, either alone or in combination with existing therapies, as the next generation of HCC therapeutics.

## 1. Introduction

Globally, liver cancer is ranked sixth in terms of cancer incidence, with approximately 800,000 new cases annually, and it ranks third in mortality rates, with an estimated 700,000 deaths annually [1–3]. It is projected that between 2020 and 2040, there will be a 55% increase in the number of new liver cancer cases, with 1.4 million people predicted to be diagnosed and 1.3 million to succumb to the disease [4]. Primary liver cancer (PLC) is a diverse group of malignant tumours with unfavourable prognoses, including hepatocellular carcinoma (HCC), intrahepatic cholangiocarcinoma (ICC), mixed hepatocellular cholangiocarcinoma, fibrolamellar HCC (FLC) and paediatric hepatoblastoma [5]. HCC is the most common PLC and ICC the second. To curb death rates, timely early diagnosis and treatment are essential in improving patient survival, and hence, regular screening for liver cancer is advised [6, 7].

Cirrhosis is the major risk factor for liver cancer, present in 80%–90% of cases, especially when hepatitis virus B (HBV) or C (HCV) is present [8]. Other risk factors for liver cancer include smoking, fatty liver disease, obesity, aflatoxin exposure and diabetes [9]. Emerging evidence also implicates gut microbiome dysbiosis and environmental pollutants, including industrial toxins and microplastics, as contributors to chronic liver inflammation and hepatocarcinogenesis [10, 11]. Furthermore, HCC affects men two to three times more frequently than it does women, and this is linked to variations in risk factors based on gender [12]. Despite the existing treatment options for liver cancer, such as liver transplant, surgery and chemotherapy, cancer recurrence remains high at more than 70% [13, 14]. In addition, and unfortunately, HCC is often detected at an advanced stage, and many patients with advanced cancer are not eligible for curative therapy [13]. Moreover, the prognosis for liver cancer is poor and is dependent on tumour stage [15].

HCC can be detected noninvasively through imaging and includes a liver protocol (dynamic 3‐phase) computed tomography (CT) scan or magnetic resonance imaging (MRI) for early arterial enhancement and delayed washout in lesions exceeding 2 cm, enhanced capsule, and growth over time and in the context of underlying hepatic disease [16]. Smaller lesions may not have evident imaging characteristics; however, elevated serum α‐fetoprotein can indicate HCC [17]. Tissue biopsy can also be performed to confirm HCC in cases where imaging is not consistent with HCC and for *de novo* lesions in the absence of underlying liver diseases [18].

To appropriately stratify patients based on prognosis and direct treatment, staging systems for HCC have been established using information about the patient and the tumour. The management of HCC and overall prognosis differ from other cancers in that they depend not only on the tumour’s characteristics but also on the liver’s underlying function, and the patient’s functional status is a significant factor in the multiple staging systems. The gold standard approach is the Barcelona Clinic Liver Cancer (BCLC) staging system. Patients are categorised as extremely early (A), intermediate (B), advanced (C), or terminal (D) based on the tumour characteristics (number and size of nodules and vascular invasion), the Child–Turcotte–Pugh score (CTP; to measure cirrhosis mortality), and the patient’s functional condition [19].

Despite improvements in diagnosis and treatment options available for liver cancer, the mortality rate is still high. Understanding liver cancer prognosis, the existing treatment options and the hepatic system is crucial in the discovery of new liver cancer drugs. Recent advances in HCC management include immune checkpoint inhibitor‐based combinations, targeted therapies and biomarker‐informed therapeutic strategies, yet durable responses remain limited by resistance, toxicity and recurrence [20–22]. In parallel, increasing evidence indicates that phytochemicals can modulate multiple oncogenic pathways relevant to HCC, but their clinical translation remains constrained by poor pharmacokinetics and tumour selectivity [23]. Nanotechnology‐based delivery systems have also emerged as a promising strategy to enhance the stability, targeting and efficacy of anticancer agents [24].

This review discusses phytochemicals that have exhibited significant activities against liver cancer and their mechanism of action and highlights new strategies and targets for treatment. The review was composed through literature searches from databases including PubMed, Google Scholar, Web of Science, PubChem and Scopus. Search terms and phrases used in the literature search included phytochemicals, liver cancer, HCC, natural compounds, phytoremedies, small molecules, signalling pathways in HCC, liver cancer treatment methods, liver cancer plant‐derived drugs at clinical trials, Food and Drug Administration (FDA)‐approved HCC plant‐derived drugs, ethnomedicines in liver cancer, liver fibrosis and cirrhosis. Relevant articles were then selected for compilation of the review. Accordingly, this review aims to (i) summarise current HCC treatment strategies and their clinical limitations; (ii) critically examine recent evidence on plant‐derived compounds with anti‐HCC activity; (iii) highlight major molecular pathways targeted by these phytochemicals; and (iv) discuss nanotechnology‐based delivery systems as a strategy to improve their translational potential.

This review is distinct and timely in that it not only summarises current therapeutic strategies for HCC but also integrates recent mechanistic evidence on plant‐derived phytochemicals with advances in nanotechnology‐based delivery systems. Linking molecular targets, translational limitations and formulation strategies, this review provides an updated framework for evaluating natural products as next‐generation adjunctive or alternative therapeutics for HCC. Unlike prior narrative summaries that focused only on phytochemicals or on HCC therapeutics, this review integrates therapeutic context, molecular pharmacology and nano‐delivery considerations into a single translational framework.

## 2. Current Treatments and Challenges

The multikinase inhibitors sorafenib and lenvatinib have been in use for HCC treatment since 2008 and 2018, respectively. Despite a modest progression‐free survival of just 3 to 5 months, sorafenib was approved by the FDA in 2007 [25–27]. The chemotherapy drug is administered orally, and its efficacy has been supported by numerous trials [28, 29]. Sorafenib can inhibit the MAP kinase cascade and suppress tumour angiogenesis and proliferation. Sorafenib has multiple protein targets, can induce apoptosis [30, 31], block vascular endothelial growth factor (VEGF) and rapidly accelerate fibrosarcoma (RAF) signalling [32], and attenuate mitochondrial function in liver cancer cells [33]. Additionally, serine–threonine kinase Raf‐1, platelet‐derived growth factor receptor β, c‐KIT, FLT‐3, VEGF receptors 2 and 3, and RET are also inhibited by sorafenib [34]. Nonetheless, 10% of patients treated with sorafenib are likely to develop cutaneous squamous cell carcinomas [35]. Moreover, studies have found that the multiple sorafenib targets are subject to genetic polymorphisms and heterogeneity that contribute to eventual resistance and treatment failure [36, 37].

Immune checkpoint inhibitors have been reported to demonstrate better efficacy than the multikinase inhibitors in HCC treatment [38]. They focus on the interaction of programmed cell death‐ligand 1 (PD‐L1) that is secreted by tumour cells and binds to programmed cell death receptor 1 (PD‐1) expressed by T‐cells and tumour‐associated macrophages to allow escape from the immune response. Atezolizumab is a humanised monoclonal IgG1 antibody that binds PD‐L1 and prevents interaction with PD‐1 receptors [39]. In 2020, atezolizumab was approved for use in conjunction with bevacizumab, a monoclonal IgG1 antibody and VEGF inhibitor, by the FDA for the treatment of HCC for patients with no prior systematic treatment. Importantly, VEGF and other immune‐evasion pathways have been connected to the onset and spread of liver cancer [40]. Thus, therapies targeting VEGF reduce VEGF‐mediated immunosuppression within the tumour and its microenvironment [41].

**FIGURE 1:**
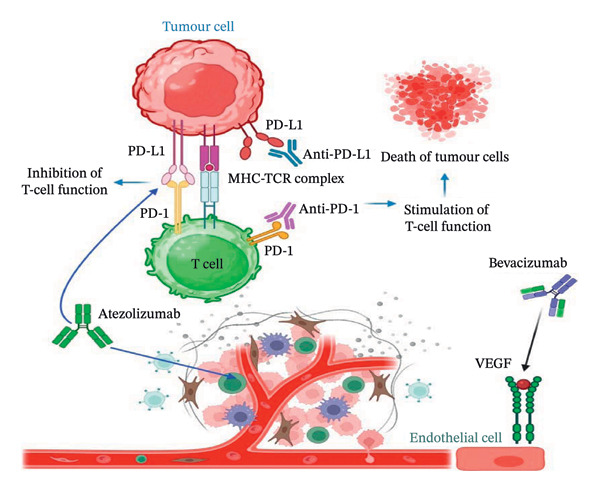
Mechanisms of action of atezolizumab and bevacizumab in HCC. Atezolizumab, an anti–PD‐L1 antibody, prevents PD‐L1 expressed by tumour and immune cells from binding to PD‐1 on CD8^+^ T‐cells, thereby counteracting immune suppression and restoring T‐cell activity. Bevacizumab, an anti–VEGF antibody, binds to soluble VEGF, preventing its interaction with VEGFR‐2 and VEGFR‐3 on endothelial cells, thereby inhibiting tumour‐driven angiogenesis and reducing VEGF‐mediated immunosuppression. Collectively, these mechanisms result in decreased tumour proliferation, inhibition of angiogenesis and enhanced antitumour immunity. The figure is adopted from [45, 46] and modified with BioRender.

The IMbrave150 clinical trial (NCT03434379) demonstrated that the combination of atezolizumab and bevacizumab improves survival outcomes in patients compared to sorafenib treatment alone. The dual therapy extended overall survival to 19.2 months, in contrast to 13.4 months for sorafenib. Furthermore, the combinatorial regimen enhanced 12‐month survival rates by 12.6% and lengthened the median progression‐free survival period to 6.9 months, compared to just 4.3 months for sorafenib [42, 43]. The efficacy of atezolizumab and bevacizumab combinatorial therapy was assessed in intermediate‐stage HCC patients and demonstrated progression‐free survival of 12.6 months, overall survival of 25.8 months, and a response rate of 44% [44]. Nonetheless, despite the significant improvement in outcomes, most patients will not achieve significant tumour shrinkage, and many relapse. Bevacizumab may also increase bleeding risks in HCC patients, and in some instances, the combinatorial therapy can promote tumour progression [39]. Figure 1 depicts key signalling pathways involved in HCC targeted by current immunotherapies, while Table 1 summarises the efficacy, side effects and therapeutic challenges. In summary, current HCC treatments have limited long‐term efficacy and significant health limitations, and together this highlights the urgent need for new therapeutic strategies.

**TABLE 1:** Comparison of current HCC therapies.

Therapy	Class/mechanism	Reported efficacy	Major side effects/limitations	Resistance/therapeutic challenge
Sorafenib	Multikinase inhibitor; inhibits MAPK cascade, tumour angiogenesis and proliferation; targets VEGF signalling and RAF signalling	Modest progression‐free survival of 3–5 months; overall survival for the comparison group in IMbrave150 was reported as 13.4 months.	Systemic toxicity; about 10% of treated patients may develop cutaneous squamous cell carcinoma; limited long‐term benefit	Genetic polymorphisms and tumour heterogeneity affecting sorafenib targets contribute to acquired resistance and treatment failure
Lenvatinib	Multikinase inhibitor used as systemic therapy for HCC	An approved treatment option for HCC since 2018.	Clinical limitations are implied as similar to targeted therapies in HCC, including incomplete durability of response	Long‐term efficacy remains limited
Atezolizumab + bevacizumab	Immune checkpoint inhibitor plus antiangiogenic therapy; atezolizumab blocks PD‐L1/PD‐1 interaction, bevacizumab inhibits VEGF	In IMbrave150, median overall survival was 19.2 months vs. 13.4 months with sorafenib; median progression‐free survival was 6.9 months vs. 4.3 months with sorafenib; improved 12‐month survival by 12.6%; in intermediate‐stage HCC, progression‐free survival was 12.6 months, overall survival was 25.8 months, response rate 44%	Bevacizumab may increase bleeding risk; many patients do not achieve major tumour shrinkage; relapse still occurs	Despite improved outcomes, many patients relapse; in some instances, therapy may be associated with tumour progression; durable responses remain limited

## 3. Natural Products in Liver Cancer Treatment

The natural world provides a near‐unlimited supply of resources for the development of new, potent medications, chemotypes and pharmacophores [47]. Medicinal plants are widely used throughout the world and have gained significant importance in the treatment of ailments. The bioactive chemicals that make up the chemical core of the plant provide a therapeutic effect [48, 49]. Natural substances have been a vital part of traditional medicine, working to both prevent and treat a wide range of illnesses [50]. Newman and Cragg established that there are 929 approved drugs of natural origin to treat various diseases, with 211 approved for antitumour management and just 35 under the category of unmodified natural products [51, 52]. For example, vinblastine and vincristine are phytochemicals from *Catharanthus roseus* approved for clinical use in cancer treatment and management [53]. In contrast, synthetic drugs are substances that have been chemically modified from naturally occurring pharmaceuticals and have the potential to have both medicinal and psychoactive effects [52]. Thus, the limited number of approved pure natural products for cancer treatment, versus the total number of approved natural‐origin drugs, suggests a need for finding more plant‐derived anticancer drugs.

A suggested source of plant‐derived drugs is from Traditional Chinese Medicine (TCM), which has a long history of treating and preventing various diseases, among them HCC. TCM treats and prevents liver diseases through several methods, including Chinese herbal medicine (CHM), acupuncture and a Chinese medicated diet [54]. Another valuable resource is network pharmacology [55], which integrates polypharmacology and network biology based on the effectiveness of highly selective single‐target medications [56]. This can be used to identify medications and disease targets from a vast quantity of data and to comprehend the molecular mechanisms in play. Network pharmacology has been applied to TCMs [57–61]. The common tools of network pharmacology include DrugBank, STITCH and TCM chemical information databases [62].

## 4. Plant‐Derived Molecules With Potential Antiliver Cancer Properties

Numerous phytochemical substances have been shown to have strong anticancer efficacy in preclinical studies. Table 2 summarises the mechanism of action of phytochemicals and the plant species from which they have been isolated, and some of these we will now consider. Ursolic acid (UA), a biologically active pentacyclic triterpene, exerts pharmacological activities such as anti‐inflammatory, anti‐angiogenic, antimetastatic and antiproliferative responses in vitro and in vivo [98]. Anticancer studies with UA have been conducted on various HCC cell lines, and it can induce apoptosis and cell cycle arrest. For example, a study with human HCC HepG2 cells demonstrated that UA promotes the release of cytochrome C from mitochondria and activates caspase‐3 to induce apoptosis [63, 99]. Tang et al. found that after treating cancer cells with UA for 24 h, the cells shrank in size, the chromatin condensed and apoptotic bodies were present [64]. Further, UA inhibited the phosphoinositide 3‐kinase (P13K)/protein kinase B (Akt) pathway that regulates cellular processes such as cell survival, cytoskeletal rearrangement and cellular apoptosis [64].

**TABLE 2:** Phytochemicals with antiliver cancer therapeutic potential.

Mechanism of action	Phytochemical	Plant source	Assay type	Key targets
Apoptosis inducers	Ursolic acid	*Vitex negundo*	In vitro	Induces apoptosis in HepG2 cells and induces inhibition of DNA replication and cell cycle arrest [63]. Inhibits P13K/Akt pathway [64].
	Homoharringtonine	*Cephalotaxus species*	In vitro, in vivo	Induces apoptosis through Hippo pathway activation [65].
	Naringin	*Citrus* seeds	In vitro	Induces apoptosis via activation of Caspase 8 and Caspase 9 [66].
	Licochalcone A	*Glycyrrhiza uralensis*	In vitro, in vivo	Upregulates Caspases‐3, 8 and 10 and downregulates Akt [67].
	Sulforaphane	*Raphanus sativus*	In vitro	Downregulates NF‐kB, inducing apoptosis in HepG2 and Hep3B cells [68].
	Asparanin A	*Asparagus officinalis*	In vitro	Induces apoptosis via upregulation of p53 [69].

PI3K/Akt pathway inhibitors	Berberine	*Coptis chinensis*	In vitro, in vivo	Inhibits PI3K/Akt pathway [70]. Downregulates NF‐κB p65 and upregulation of miR‐22‐3p [71].
	Baicalein	*Scutellaria baicalensis*	In vitro	Inhibits the PI3K/Akt signalling pathway [72].
	Hesperidin	*Citrus seeds*	In vivo	Downregulates the PI3K/Akt pathway [73].
	Oleuropein	*Istrska belica*	In vitro	Induces apoptosis via downregulation of the PI3K/Akt pathway [74].
	Apigenin	*Matricaria chamomilla*	In vitro, in vivo	PI3K/Akt/mammalian target of rapamycin (mTOR) signalling pathway inhibition [75].

MAPK/STAT3 modulators	Scutellarin	*Scutellaria barbata* and *Scutellaria lateriflora*	In vitro, in vivo	Induces apoptosis of HepG2 cells via the STAT3 signalling pathway [76]. Downregulates STAT3/Girdin/Akt signalling pathway [77].
	Quercetin	*Allium cepa*	In vitro, in vivo	Regulates the Janus kinase 2 (JAK2)/STAT3 pathway and inhibits PI3K/Akt and ERK pathways [78].
	Gallic acid	*Punica granatum*	In vivo	Regulates the STAT3 pathway [79].
	Cepharanthine	*Stephania cepharantha*	In vitro	Downregulates Akt pathway and activation of JNK1/2 signalling pathway [80].
	Hispidulin	*Salvia involucrata*	In vitro, in vivo	Inhibits the PI3K/Akt pathway [81]. Upregulation of PPARγ [82].
	Luteolin	*Ixeris sonchifolia*	In vitro	Suppresses activity of NF‐kB [83].

ROS modulators	Curcumin	*Curcuma longa*	In vitro, in vivo	Decreases HIF‐1 and VEGF expression and regulates PI3K/Akt/mTOR signalling pathway [84–86].
	Resveratrol	*Veratrum grandiflorum*	In vitro	Inhibits TNF‐α‐mediated MMP‐9 expression via downregulation of NF‐κB activity and blockade of cell invasion in HCC cells [87].
	Emodin	*Rheum palmatum*	In vitro	Upregulates miR‐34a and inhibits VEGFR_2_ [88]. Induces apoptosis via triggering of mitochondrial‐mediated ROS generation [89].

Anti‐angiogenic/antimetastatic	Kaempferol	*Kaempferia galanga*	In vitro, in vivo	Inhibits MAPK and HIF‐1 activity [90].
	Matrine	*Sophora flavecens*	In vivo	Increases miR199a‐5p expression [91]
	Nitidine chloride	*Zanthoxylum nitidum*	In vitro	Suppresses activation of ERK, STAT3 and SHH pathways and alters CDK4, Bcl‐2, Cyclin D1, VEGF‐A and VEGFR2 expression [92].

Epigenetic/metabolic modulators	Chlorogenic acid	*Etingera elatior*	In vitro, in vivo	Inhibits DNMT1 expression [93].
	Pterostilbene	*Pterocarpus santalinus*	In vitro	Downregulates mTOR, STAT3, S6K1 and p53 expression [94].
	Capsaicin	*Capsicum* species	In vitro	Regulates the SIRT1/NOX4 signalling pathway [95].
	Berbamine	*Berberis amurensis*	In vitro, in vivo	Inhibits expression of Ca2+/calmodulin‐dependent protein kinase II [96].
	Icaritin	*Epimedium sagittatum*	In vitro, in vivo	Inhibits SphK activity and activation of p53 [97].

*Note:* All the above compounds are at the preclinical level for HCC.

Nitidine chloride is a bioactive compound commonly isolated from *Zanthoxylum nitidum*, a traditional Chinese medicinal herb, and can inhibit the growth of cancer cells, including HCC, by inducing apoptosis [100]. Nitidine chloride also suppressed the activation of extracellular signal‐regulated kinases (ERK), signal transducer and activator of transcription 3 (STAT3) and sonic hedgehog (SHH) pathways and the expression of cyclin‐dependent kinase 4 (CDK4), B‐cell lymphoma 2 (Bcl‐2), cyclin D1 (CCND1), VEGF‐A and VEGFR2. It is safe, with no nephrotic or hepatic damage observed at a dose of 10 mg/kg in mice [101].

Homoharringtonine is a natural alkaloid isolated from *Cephalotaxus* and has been used in TCM for many decades, especially for treating leukeamia. It has inhibitory activity against HCC cell lines, including HepG2, SMMC7721, Hep3B, Bel‐7402 and Bel‐7404 [101]. Wang et al. established that homoharringtonine induces apoptosis through the activation of the Hippo pathway [65]. Scutellarin, a flavonoid that has been isolated from many medicinal plants, including *Scutellaria barbata* and *Scutellaria lateriflora*, exhibits various pharmacological activities, among them anticancer [102]. Scutellarin inhibited the proliferation of HepG2 cells in a concentration and time‐dependent manner, promoted the generation of reactive oxygen species (ROS) and induced apoptosis via the STAT3 signalling pathway [76, 103].

Kaempferol, a flavonoid commonly extracted from fruits and vegetables, demonstrates various health‐beneficial effects in chronic diseases, such as cancer [104]. Kaempferol modulates signal transduction pathways related to apoptosis, cell cycle arrest, angiogenesis, inflammation and metastasis [105]. Kaempferol inhibits mitogen‐activated protein kinase (MAPK) and HIF‐1 activity at physiologically relevant doses and effectively suppresses HCC cell viability under hypoxia [106]. Matrine, an alkaloid isolated from medicinal plants such as *Sophora flavecens*, is reported to demonstrate potential capacity as an HCC drug. Matrine has a substantial antimetastatic effect in HCC by increasing miR199a‐5p expression, which inhibits HIF‐1α signalling and epithelial mesenchymal transition (EMT), suggesting miR199a‐5p is a possible therapeutic target [91]. Additionally, matrine suppressed cell growth and HCC carcinogenesis by elevating miR‐345‐5p and decreasing circular RNA (circ_0027345) and homeobox‐containing D3 (HOXD3) expression [107].

Berberine, an isoquinoline alkaloid, has been used to treat various diseases [108], such as inflammatory, hypolipidemic, viral, cancerous, hypotensive and hypoglycaemic disorders [109–113]. Berberine, isolated from *Coptis chinensis,* exhibits strong anticancer properties by inhibiting cell proliferation and induces apoptosis through AMP‐activated protein kinase (AMPK), Akt and MAPK [114]. The treatment of Hep3B and Bel‐7404 cells with berberine decreased the uptake of glutamine by inhibiting solute carrier family 1 member 5 (SLC1A5), leading to suppressed cell proliferation [115]. The combination of S‐allyl‐cysteine and berberine can reduce necroptosis and proliferation via regulating adenylate cyclase signalling and stabilising mitogen‐activated protein kinase 3/6 (MKK3/6) [116]. Chen et al. established that berberine can inhibit HCC cancer cell growth via the regulation of specificity protein 1 (Sp1) and miR‐22‐3p and their downstream targets Bcl‐2 and CCND1 [117]. Additionally, berberine therapy can restrict cell growth by suppressing nuclear factor kappa B (NF‐κB) p65 to induce apoptosis and targeting Sp1 to upregulate miR‐22‐3p [71]. Berberine also enhances ROS production, increases apoptosis and inhibits HCC cell growth through inhibiting glutamic‐pyruvic transaminase 1 (GPT1) [118, 119]. PI3K/Akt pathway inhibition by berberine inhibits cell growth, invasion and migration and induces apoptosis. Further, a combination of berberine and HMQ1611 inhibits the WNT/β‐catenin pathway and HCC proliferation and metastasis [70, 120].

Curcumin, isolated from *Curcuma longa,* inhibits various processes, including inflammation, tumour growth and oxidation and viral infection such as HIV [121]. In HCC curcumin was found to induce apoptosis in HepG2 cells, as indicated by DNA laddering, chromatin condensation and fragmentation. Mechanistically, curcumin treatment decreased p53 expression while Bcl‐2 levels were unchanged, suggesting that other p53‐dependent survival mechanisms are in play [122]. Curcumin also decreased HCC tumour growth by inhibiting HIF‐1 and degrading the aryl hydrocarbon receptor nuclear translocator (ARNT) [123].

Resveratrol, a polyphenolic phytoconstituent, has demonstrated biological activity in various diseases. In HCC, resveratrol inhibited proliferation of HepG2 cells [124] and upregulated Bcl‐2‐associated X protein (Bax), downregulated Bcl‐2 and activated the p53 pathway [125]. Further, resveratrol inhibited TNF‐mediated invasion of HepG2 cells, arrested H22 cell growth, and exhibited a synergistic effect when mixed with 5‐fluorouracil (5‐FU) to induce apoptosis [87].

Icaritin, a flavonoid isolated from various medicinal plant species, has demonstrated biological anticancer and anti‐inflammatory activities [126]. Icaritin suppressed cell growth and induced cell apoptosis and reduced alpha‐foetal protein (AFP) expression in HepG2 and SMMC7721 cells. AFP can induce tumour cell proliferation and inhibit apoptosis; thus, its suppression may promote the reverse [97]. In sum, the phytochemicals discussed in this section have exhibited activity against liver cancer.

Together, the many phytochemicals presented have clear anti‐HCC activity, and a clear pattern emerges, with consistent targeting of apoptosis pathways and key oncogenic signalling cascades, including PI3K/Akt, MAPK, STAT3 and NF‐κB. Despite this consistency, the evidence remains largely preclinical, with significant variability in experimental models, compound purity and dosing strategies, limiting direct comparisons across studies. Importantly, many compounds such as berberine and curcumin exhibit strong mechanistic efficacy but fail to translate clinically due to poor bioavailability, rapid metabolism and insufficient tumour targeting. These limitations highlight the need for integrative strategies that combine mechanistic insights with optimised delivery systems to bridge the gap between preclinical promise and clinical application.

## 5. Structural Features Underpinning the Anticancer Activity of Phytochemicals

The natural phytochemicals such as berberine, curcumin, resveratrol and icaritin exhibit considerable anticancer activity against HCC through diverse and complex molecular mechanisms. The low toxicity, multitargeted mechanisms and abundant bioavailability of phytochemicals render them as promising candidates for cancer therapy. These compounds modulate critical signalling pathways, including AMPK, Akt, MAPK, NF‐κB and WNT/β‐catenin, while also regulating apoptosis‐related genes and metabolic processes. Their ability to inhibit tumour proliferation, induce apoptosis, suppress invasion and metastasis, and enhance chemosensitivity underscores their potential as valuable adjuncts to conventional liver cancer therapies. Nonetheless, despite compelling preclinical evidence, the clinical application of these compounds remains limited, primarily due to challenges related to bioavailability, pharmacokinetics and dosage optimisation. Continued research, including well‐designed clinical trials and advanced drug delivery strategies, is essential to fully harness the therapeutic potential of these and other potential plant‐derived agents in the management of liver cancer.

**FIGURE 2:**
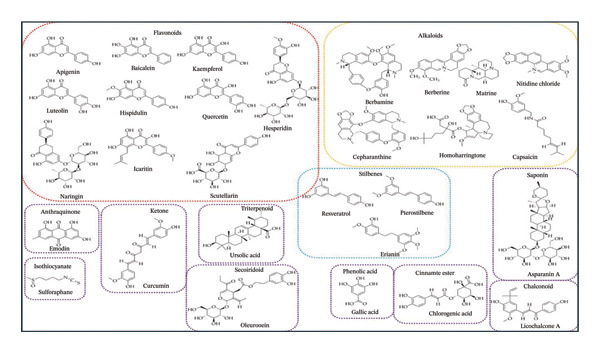
Chemical structures of isolated phytomolecules with anticancer properties. Structural classification of phytochemicals highlights flavones and alkaloids as predominant classes. Both classes feature planar, highly conjugated scaffolds, flavones with a flavone backbone and alkaloids with indole or isoquinoline rings, enabling intercalation into DNA or binding to kinase ATP pockets. Key functional groups such as hydroxy, carbonyl and heteronitrogen moieties facilitate hydrogen bonding within enzymatic active sites, including Topoisomerase I/II, MAPKs and PI3K, potentially underpinning their anticancer activities.

The structural classification of the phytochemicals discussed in this article revealed that flavones and alkaloids are a majority (Figure 2). The analysis of these two main chemical classes indicates shared elements that could be contributing to their anticancer activities. Both have a planar, highly conjugated scaffold that is the flavone backbone and indole or isoquinoline rings that could slip between DNA base pairs or stack in kinase ATP pockets, blocking transcription, replication or signalling [127]. The well‐placed hydroxy groups or carbonyls in the flavones and hetero‐nitrogen in the alkaloids, which are hydrogen‐bond donors/acceptors, have the potential to anchor the molecule inside enzymatic active sites, such as topoisomerase I/II, MAPKs and P13K [128, 129]. The redox‐active phenolics and nitrogen heterocycles could modulate cellular ROS to tip cancer cells into apoptosis. In the case of triterpenoids, the hydroxy group at position 3 and the carboxylic acid (position 28) have the potential to enhance binding to the NF‐κB/inhibitor of kappa B (IκB) complex to stimulate caspase activation and to inhibit STAT3 and mTOR. Together, these chemical attributes may have purpose in developing new liver cancer therapies.

## 6. Nanotechnology Approach in Phytoremedies Delivery

Nanotechnology‐based drug delivery systems have demonstrated substantial improvements in therapeutic efficacy, particularly in oncology [130]. In HCC, nanocarrier‐mediated drug delivery has been shown to reduce systemic toxicity, lower the required therapeutic dose, prolong sustained drug release following a single administration, and enhance selective targeting of malignant cells [131–134]. Organic dye‐doped, luminescent core–shell nanoparticles covalently conjugated to human liver cancer monoclonal antibodies have demonstrated high photostability and selective targeting of HepG2 cells in vitro, with minimal dye leakage. Similarly, docetaxel encapsulated within rattle‐type mesoporous silica nanoparticles exhibited enhanced cytotoxicity against HepG2 cells, achieving comparable therapeutic outcomes using only 70% of the free drug dose, alongside reduced systemic toxicity and a 15% improvement in tumour inhibition in hepatocarcinoma‐bearing mice [135]. Complementary findings were reported by Hong and colleagues, who demonstrated a 70%–80% dose reduction using doxorubicin‐loaded microspheres compared with conventional intra‐arterial administration [136].

Nanotechnology‐based drug delivery strategies have also progressed to clinical evaluation for HCC. Clinical trials involving NBTXR3, a hafnium oxide crystal nanoparticle formulation activated by stereotactic body radiation therapy (SBRT), have demonstrated therapeutic potential when administered 24 h prior to irradiation [137]. Additionally, superparamagnetic iron oxide nanoparticles (SPIONs) have emerged as effective HCC drug carriers due to their capacity for external magnetic field‐guided targeting [132, 138]. Multiple useful carrier platforms, including polymeric nanoparticles, solid lipid nanoparticles, nanostructured lipid carriers, liposomes, niosomes, dendrimers and micelles for compounds such as curcumin, resveratrol, quercetin and berberine, have been evaluated [139]. For example, curcumin‐loaded PLGA nanoparticles, liposomal curcumin formulations, and hepatocyte‐targeted galactosylated chitosan–polycaprolactone curcumin nanoparticles have shown improved stability, delivery and anticancer performance. While berberine nanoformulations, including liposomal, chitosan, PLGA and self‐nanoemulsifying systems, have demonstrated enhanced oral absorption, sustained delivery, and stronger antitumour effects than free berberine [140].

Despite these advances, the clinical translation of phytochemical nanomedicines remains challenging. Key barriers include scalable and reproducible manufacturing, batch‐to‐batch consistency, complex physicochemical characterisation, possible immune recognition and altered biodistribution, uncertain long‐term toxicity or tissue accumulation, and regulatory hurdles that slow translation from preclinical studies to clinical use. Thus, while nanoformulations may address the major pharmacokinetic limitations of phytochemicals, their therapeutic promise must be interpreted alongside these translational constraints [141].

Collectively, these nanotechnology platforms present significant opportunities for the targeted delivery of plant‐derived therapeutics, potentially enhancing the efficacy, bioavailability and clinical translation of phytomedicines. Emerging strategies such as stimuli‐responsive nanoparticles and exosome‐based delivery systems offer additional opportunities to enhance targeted delivery and therapeutic efficacy of phytochemicals in HCC [142, 143].

## 7. Conclusion

HCC remains one of the most lethal malignancies worldwide and continues to pose a growing global health burden. Current projections indicate that, if incidence and mortality trends remain unchanged, liver cancer cases and associated deaths will increase by more than 50% over the next two decades. Despite notable advances in diagnosis and systemic therapies, clinical outcomes remain unsatisfactory, largely due to late‐stage diagnosis, high recurrence rates, treatment‐related hepatotoxicity and cardiotoxicity, the emergence of drug resistance, and limited long‐term efficacy of existing therapeutic regimens.

This review highlights the substantial therapeutic potential of natural products, particularly plant‐derived phytochemicals, as complementary or alternative strategies for HCC management. A wide range of phytochemicals, including flavonoids, alkaloids, triterpenoids and polyphenols, demonstrate robust anti‐HCC activity through multitarget mechanisms involving apoptosis induction, cell cycle arrest, inhibition of angiogenesis and metastasis, modulation of oxidative stress, and suppression of oncogenic signalling pathways such as PI3K/Akt, MAPK, STAT3, NF‐κB and HIF‐1. Importantly, many of these compounds exhibit comparatively favourable toxicity profiles, underscoring their suitability for long‐term or combinatorial therapeutic strategies.

In addition to their standalone anticancer effects, phytochemicals show promise as chemosensitizers that enhance the efficacy of conventional therapies and potentially mitigate treatment‐associated toxicity. Advances in nanotechnology‐based drug delivery systems further address key translational barriers by improving solubility, bioavailability, tumour selectivity and pharmacokinetic stability of phytochemicals. The convergence of phytochemistry, nanomedicine, and emerging computational approaches offers a powerful framework for accelerating the discovery and clinical translation of plant‐derived therapeutics.

While multiple phytochemicals consistently target apoptosis, PI3K/Akt, MAPK, STAT3, NF‐κB, HIF‐1, and metastatic pathways, most evidence remains preclinical and has not yet translated into standardised clinical interventions. Critical challenges include insufficient pharmacokinetic characterisation, variability in phytochemical composition, poor bioavailability, a lack of standardised formulations, and a paucity of rigorously designed clinical trials. In parallel, nanotechnology‐based delivery systems, although effective in enhancing drug stability and targeting, encounter challenges related to large‐scale manufacturing, potential immunogenicity, long‐term safety concerns, and regulatory approval pathways. Furthermore, most evidence supporting both approaches remains preclinical, with limited well‐designed clinical trials validating their efficacy in HCC patients. Addressing these limitations will be critical for advancing phytochemical‐based nanomedicine into clinical practice. Combinatorial strategies integrating phytochemicals, nanocarriers and existing systemic therapies may provide a synergistic approach to overcoming drug resistance and improving therapeutic outcomes in HCC.

**Nomenclature**
5‐FU: 5‐fluorouracil
Akt: Protein kinase B
BCLC: Barcelona Clinic Liver Cancer
CHM: Chinese herbal medicine
CT: Computed tomography
CTP: Child–Turcotte–Pugh
CDK4: Cyclin‐dependent Kinase 4
EMT: Epithelial mesenchymal transition
ERK: Extracellular signal‐regulated kinases
FDA: Food and Drug Administration
FLC: Fibrolamellar HCC
HBV: Hepatitis virus B
HCC: Hepatocellular carcinoma
HCV: Hepatitis virus C
HOXD3: Homeobox‐containing D3
ICC: Intrahepatic cholangiocarcinoma
JAK: Janus kinase
MAPK: Mitogen‐activated protein kinase
MRI: Magnetic resonance imaging
mTOR: Mammalian target of rapamycin
PD‐1: Programmed cell death Receptor 1
PD‐L1: Programmed cell death‐Ligand 1
PLC: Primary liver cancer
RAF: Rapidly accelerated fibrosarcoma
ROS: Reactive oxygen species
STAT3: Signal transducer and activator of Transcription 3
TCM: Traditional Chinese medicine
UA: Ursolic acid
VEGF: Vascular endothelial growth factor

## Author Contributions

Conceptualization, Phurpa Wangchuk and Felix Wambua Muema; writing–original draft preparation, Felix Wambua Muema.; writing–review and editing, Phurpa Wangchuk, Lionel Hebbard and Catherine M. Miller; funding acquisition, Phurpa Wangchuk, Catherine M. Miller and Lionel Hebbard. All authors have read and agreed to the published version of the manuscript.

## Funding

This study was supported by the Tropical Australian Academic Health Centre, SF0000121; Townsville Hospital and Health Service—Study Education Research Trust Account, THHSSERTA_RPG1 2023; NHMRC Ideas Grant, APP1183323; International Research Training Program Stipend (IRTPS) by Australian Government.

## Conflicts of Interest

The authors declare no conflicts of interest.

## Data Availability

This review article does not include any original data or new analysis. All information presented is derived from previously published studies cited throughout the manuscript.
